# Dose-Dependent Effects of Heat Shock Cognate 70 on Viability and Apoptosis-Related Gene Expression in In Vitro-Produced Bovine Embryos

**DOI:** 10.3390/vetsci13040339

**Published:** 2026-03-31

**Authors:** Aimé Jazmin Garza-Arredondo, Diana Eliza Zamora-Ávila, Gustavo Moreno-Degollado, Denisse Melisa Garza Hernandez, José Fernando de la Torre Sanchez, Sandra Pérez-Reynoso, Rubén Cervantes-Vega, Uziel Castillo-Velázquez

**Affiliations:** 1 Cuerpo Académico de Nutrición y Producción Agroalimentaria, Facultad de Medicina Veterinaria y Zootecnia, Universidad Autónoma de Nuevo León, General Escobedo 66054, Mexico; aime.garzaarr@uanl.edu.mx (A.J.G.-A.); diana.zamoravl@uanl.edu.mx (D.E.Z.-Á.); gustavo.morenod@uanl.mx (G.M.-D.); dgarzahr@uanl.edu.mx (D.M.G.H.); ruben.cervantesvg@uanl.edu.mx (R.C.-V.); 2 Centro Nacional de Recursos Genéticos, Instituto Nacional de Investigaciones Forestales, Agrícolas y Pecuarias (INIFAP), Tepatitlán de Morelos, Jalisco 47600, Mexico; jose.delatorre@academicos.gob.mx (J.F.d.l.T.S.); perez.sandra@inifap.gob.mx (S.P.-R.); 3 Facultad de Medicina Veterinaria y Zootecnia, Campus de Ciencias Agropecuarias, Universidad Autónoma de Nuevo León, Ave. Francisco Villa s/n, Ex-Hacienda El Canadá, General Escobedo 66050, Mexico

**Keywords:** bovine, blastocyst, HSC70, apoptosis, gene expression

## Abstract

Endogenous heat shock cognate 73 kDa protein (HSC70) plays a role in early embryonic development and cellular stress regulation. This study evaluated the effects of exogenous recombinant HSC70 supplementation on bovine embryo development and the expression of apoptosis-related genes under *in vitro* conditions. Expression analyses of HSPA1A, HSPA8, *BCL-2*, and *BAX* were performed on Day 7 bovine embryos produced *in vivo* and *in vitro*. In vitro embryos exhibited higher basal expressions of HSPA8, *BAX* and *BCL-2* compared with in vivo embryos (*p* ≤ 0.001). Supplementation with 500 or 1000 ng/mL HSC70 was associated with increased expression of *HSPA1A*, *HSPA8*, *BCL-2*, and *BAX* relative to control embryos (*p* ≤ 0.01). The 1000 ng/mL group showed significantly higher *HSPA8* expression compared with both the control and 500 ng/mL groups. Morphological evaluation indicated that embryos supplemented with 500 ng/mL were associated with improved blastocyst yield and quality compared with control and 1000 ng/mL groups.In conclusion, supplementation with 500 ng/mL recombinant HSC70 was associated with modulation of apoptosis-related gene expression and improved morphological developmental parameters under in vitro conditions. These findings indicate dose-dependent regulatory effects of HSC70 on apoptosis-related signaling pathways; however, as apoptosis was assessed at the transcriptional level, the results should be interpreted as molecular associations rather than direct confirmation of altered apoptotic activity.

## Simple Summary

Heat shock proteins (HSPs) help embryos cope with stress during early development. In this study, we evaluated whether supplementation with recombinant HSC70 modulates apoptosis-related gene expression and morphological development in bovine embryos cultured *in vitro*. A low dose (500 ng/mL) was associated with increased expression of anti-apoptotic genes and improved morphological blastocyst quality. In contrast, a higher dose (1000 ng/mL) was associated with increased expression of a pro-apoptotic gene and reduced blastocyst formation. These observations are based on transcriptional and morphological assessments and suggest that appropriate levels of HSC70 may influence early embryo development in cattle.

## 1. Introduction

*In vitro* embryonic development is regulated by multiple factors, including the origin of gametes, culture conditions, microenvironmental characteristics, and exposure to environmental stressors. During post-fertilization culture, critical developmental events occur that determine blastocyst quality [1]. However, embryos cultured in vitro are exposed to stress conditions not typically encountered within the bovine reproductive tract [2]. Exogenous factors such as light exposure, elevated oxygen tension, and culture medium composition may induce metabolic stress and increase reactive oxygen species (ROS) production [3], leading to lipid peroxidation, protein modification, DNA fragmentation, and apoptosis, ultimately impairing embryo development [4,5].

Apoptosis is an essential programmed cell death pathway in the development process. It eliminates redundant or superfluous cells to allow for normal patterning to proceed [6]. Irreparably stressed and damaged cells also use this route for removal. Pathological stimuli, such as radiation, chemotherapy, and environmental toxicants, can initiate apoptosis in oocytes [7,8,9]. Heat shock can also induce apoptosis in pre-implanted embryos [10,11,12].

To improve the developmental proficiency of pre-implanted blastocysts, oocyte maturation and in vitro culture media have been supplemented with antioxidants, such as peroxiredoxin II [13], resveratrol [14,15], green tea polyphenols [16] and melatonin [17,18]. This enhances the quality of the embryos through the reduction in ROS, increased expression of anti-apoptotic genes (*BCL-2*), and decreased expression of pro-apoptotic genes (*BAX* and caspase-3).

Moreover, molecular chaperones protect cells from damage caused by physical and chemical hazards, such as increased temperature (e.g., heat shock), anoxia, hypoxia, metabolic stress-associated cytokines, nitrogen oxide, ethanol, heavy metals, apoptosis-inducing agents, and other chemical denaturants and drugs [19,20]. Chaperones, such as the heat shock proteins (HSPs), play an important role in facilitating protein folding and maintaining normal protein structure and function [21].

HSPs are primarily classified based on their molecular weight in kilodaltons (kDa) as HSP110, HSP100, HSP90, HSP70, HSP60, HSP40, and the small HSP family (20 to 25 kDa). Within the HSP70 family, constitutively expressed, HSC70 (73 kDa), is slightly upregulated during stress conditions. Unlike the inducible HSP70 isoform, which is primarily expressed in response to cellular stress, HSC70 is constitutively expressed and plays a central role in basal proteostasis and protein quality control. Due to its continuous expression during early embryonic stages, HSC70 represents a biologically relevant candidate for investigating regulatory effects on embryonic stress adaptation and apoptosis-related pathways. Heat shock cognate protein 70 (HSC70) is particularly abundant in mammalian embryos [22]. It participates in the uptake of proteins into the nucleus and other cellular organelles, such as the endoplasmic reticulum and mitochondria, and maintains the translocation-competent state of proteins destined for these locations [23,24,25]. HSC70 maintains protein homeostasis during normal and stress conditions, suppresses protein aggregation, and reactivates heat-denatured proteins [21]. Because of these protective functions associated with HSC70 and the low percentage of embryos produced *in vitro*, we evaluated the effect of HSC70 supplementation on bovine embryo viability *in vitro*.

## 2. Materials and Methods

### 2.1. Animals

Simbrah cows (*n* = 6) were maintained under standard managerial practices in Centro de Investigación y Producción Agropecuaria de la Universidad Autónoma de Nuevo León (Linares, Mexico) were used for in vivo embryo production.

Experiments were performed in accordance with Facultad de Medicina Veterinaria y Zootecnia de la Universidad Autónoma de Nuevo León guidelines for the care and use of animals (approval number 01/2021). Maturation (IVM), fertilization (FCDM), early culture (CDM1), and late culture (CDM2) media were supplemented with fatty acid-free bovine serum albumin prepared at the National Center for Genetic Resources of the National Institute for Agricultural and Livestock Forestry Research (Tepatitlán de Morelos, Jalisco, Mexico) according to data published by De La Torre Sanchez et al. [26].

### 2.2. In Vivo Embryo Production

Estrus was synchronized using an intravaginal progesterone-releasing device (CIDR) (Pfizer, New York, NY, USA) for 7 days. Cows were treated with 2.0 mg i.m. of Syntex estradiol benzoate (Zoetis, Mexico City, Mexico) at the time insertion of the CIDR device (day 0). On days 4, 5, and 6, 360 mg i.m. of Folltropin-V, follicle-stimulating hormone (Bioniche, Rockford, IL, USA) was administered. On day 6, the cows were treated with two doses (25 mg i.m. every 12 h) of dinoprost tromethamine (Lutalyse, Zoetis, Mexico City, Mexico). On day 7, the CIDR withdrawal time, another dose of follicle-stimulating hormone was administered. Artificial insemination was performed twice on day 8, in the morning and afternoon.

Seven days after artificial insemination, the embryos were recovered using two-way Foley catheters (Nipro Medical Corporation, Bridgewater, NJ, USA). ViGRO complete flush solution (Bioniche, Rockford, IL, USA) was introduced and the medium containing the embryos was obtained. Embryos were washed using the same solution and placed into Petri dishes. A stereoscopic microscope-assisted search for embryos and identification (stage of development and quality of embryos) was performed based on the Manual of the International Embryo Technology Society (IETS) guidelines (Savoy, IL, USA, 2010). Subsequently, blastocysts were stored in pools of three per Eppendorf tube with 100 μL Trizol Reagent (Invitrogen, Carlsbad, CA, USA) at 5 °C until RNA extraction and real-time quantitative reverse transcription (qPCR) analysis.

### 2.3. Oocyte Retrieval and In Vitro Maturation

Ovaries were obtained from the local abattoir and transported to the laboratory within 2 h in physiological saline (0.9% NaCl). Cumulus–oocyte complexes were recovered from follicles at a diameter of 3–6 mm using an 18-gauge needle attached to a 10-mL syringe. Follicular fluid and the cell package were placed into a 50 mL conical tube at 38.5 °C. Selected cumulus–oocyte complexes with a uniform granular cytoplasm and surrounded by a multiple compact mass of cumular cells, were cultivated in IVM medium for 23 h at 38.5 °C in a humidified atmosphere of 5% CO_2_ in air. The medium included 2 µL/mL FSH (NIDDK-o FSH-20), 1 µL/mL LH (NIH-LH-S1), 5 µL/mL hCG (10,000 IU), 10 µL/mL β-estradiol (1 mg), cysteamine (0.1 mM), and 1 µL/mL epidermal growth factor (0.5 mg) (Sigma–Aldrich, St. Louis, MO, USA) based on previously published data [27].

### 2.4. In Vitro Fertilization and Culture

Mature oocytes (*n* = 600) were transferred to FCDM medium for co-culture with spermatozoa. Semen was thawed and processed using a Percoll gradient (Sigma–Aldrich, St. Louis, MO, USA) (45:90%). Mature oocytes and sperm were incubated together at 5% CO_2_ in humidified air at 38.5 °C for 18 h prior to *in vitro* culture.

After co-incubation, cumulus cells were removed using a pipette. The presumptive zygotes were washed, randomly divided (*n* = 160 per group), and transferred to 100 µL CDM1 medium supplemented with HSC70 (Sigma–Aldrich, St. Louis, MO, USA) at 500 or 1000 ng/mL. In addition, a control group was established without supplementation. The samples were incubated for 56 h at 38.5 °C in a humidified atmosphere of 5% CO_2_, 5% O_2_, and 90% N_2_ in air. For blastocyst cell number assessment, Day 7 blastocysts were subjected to differential staining according to Thouas et al. [28], and the total number of cells was subsequently determined.

### 2.5. Embryo Culture Conditions o HSC70 Supplementation

Recombinant bovine HSC70 (Sigma-Aldrich, St. Louis, MO, USA; Product No. H8285) was expressed in *Escherichia coli* and supplied as a buffered aqueous solution with a reported purity ≥ 90% (SDS-PAGE). Stock solutions were stored at −70 °C according to the manufacturer’s recommendations.

The concentration range was selected to evaluate dose-dependent regulatory effects on embryonic stress response and apoptosis-related pathways. Lower concentrations were intended to approximate physiological modulation of chaperone-mediated proteostasis, whereas higher concentrations were included to assess potential threshold or saturation effects under *in vitro* culture conditions.

Embryos were transferred and cultured in CDM2 medium and supplemented with HSC70 (Sigma–Aldrich, St. Louis, MO, USA) (500 and 1000 ng/mL) or not (control group) according to the group in which they previously belonged. For gene expression assays, embryos were maintained for 48 h. For morphological analysis, embryos were maintained in culture for 120 h under the conditions described above. At the end of the culture period, blastocysts were identified and classified according to IETS manual numerical codes corresponding to the developmental stage and quality.

### 2.6. RNA Extraction Using a Modified Trizol Method and Complementary DNA (cDNA) Synthesis

Using a modified Trizol protocol [29], RNA was isolated from three biological replicates. Embryos (≥8 cells, in pools of three) were added to Eppendorf tubes with 100 μL Trizol (Invitrogen, Carlsbad, CA). The samples were incubated at room temperature for 3 min. Next, 50 µL chloroform was added, the samples were inverted for 15 s, and then centrifuged at 12,000 *g* for 30 min at 4 °C. The aqueous phase was transferred to a fresh tube and RNA was precipitated with the addition of 2.5 volumes of isopropanol. Following centrifugation at 12,000 *g* for 30 min at 4 °C, the supernatant was discarded, the pellet was washed with 50 µL 70% ethanol, and centrifuged at 7500 *g* for 5 min. Finally, the pellet was dried in an incubator for 30 min at 37 °C and dissolved in 20 µL of DEPC water.

The eluted RNA was reverse-transcribed into cDNA using the GoTaq Probe 2-step qRT-PCR System kit (Promega, Madison, WI, USA). Briefly, 5 µL RNA, 1 µL oligo dT (0.5 mg/µL), and 1 µL of random primer were added to each tube. Subsequently, the tubes were incubated in a heat block at 70 °C for 5 min and immediately placed on ice for at least 5 min. Then, 4.9 µL nuclease-free water, 4 µL GoScript 5× Reaction Buffer, 1.6 µL MgCl_2_, 1 µL dNTPs, 0.5 µL Recombinant RNasin Ribonuclease Inhibitor, and 1 µL GoScript Reverse Transcriptase were combined and incubated 25 °C for 5 min. Subsequently, the reaction was extended in a controlled-temperature heat block at 42 °C for 45 min and inactivated at 70 °C for 15 min. The resulting complementary DNA was quantified using a Quantus fluorometer (Promega, Madison, WI, USA) and stored at −20 °C until use.

For gene expression analysis, embryos were pooled in groups of three. Each pool originated from a single independent experimental replicate and was considered one biological replicate for statistical analysis (*n* = 6 per group). Donor animals were not treated as independent experimental units.

### 2.7. Quantitative Real-Time PCR Analysis

Real-time RT-PCR was performed with an Applied Biosystems 7500/7500 Fast detection system (Thermo Fisher Scientific, Waltham, MA, USA). All primers and probes used were designed by Integrated DNA Technologies (Coral Ville, IA, USA). PrimeTime probes were synthesized and labeled with HEX and FAM (Table 1). The measurement of *HSP1A, HSPA8, BAX,* and *BCL-2* mRNA was done in triplicate using the GoTaq Probe 2-step qRT-PCR System kit (Promega, Madison, WI, USA), in a 20 μL reaction mixture containing GoScript 5× reaction buffer, 25 mM MgCl2, 10 mM dNTP mix, 40 u/µL Recombinant RNasin ribonuclease inhibitor, GoScript Reverse Transcriptase, 500 µg/mL random primer, ROX, and 250 nM of probe.

The amplification conditions for all genes were as follows: 50 °C for 2 min, 95 °C for 10 min, followed by 40 cycles of 95 °C for 15 s, 60 °C for 15 s, and 60 °C for 15 s. The gene results were analyzed according to the comparative ΔΔCt method and are reported as relative expression or fold-difference in expression to a housekeeping gene (*GAPDH*).

### 2.8. Statistical Analysis

Gene expression data were analyzed according to data distribution. Normality was assessed prior to analysis. When normal distribution assumptions were met, one-way analysis of variance (ANOVA) followed by appropriate multiple comparison tests was applied. For non-normally distributed data, non-parametric tests were used. Comparisons between in vivo and in vitro embryos were performed using Dunn’s post hoc test. Differences among embryo quality categories and between HSC70-supplemented and control groups were evaluated using Kruskal–Wallis tests followed by Bonferroni-adjusted multiple comparisons when appropriate.

Developmental outcomes, including cleavage rate, proportion of embryos reaching the ≥6-cell stage, blastocyst formation on Days 6 and 7, and total blastocyst cell number, were analyzed using one-way ANOVA followed by Tukey’s multiple comparison test. Percentage data were calculated per independent experimental replicate prior to statistical comparison. Each replicate (*n* = 6 independent experimental replicates) was considered the experimental unit.

Data are presented as mean ± SEM. Statistical significance was established at *p* < 0.05. GraphPad Prism version 4.0 (GraphPad Software Inc., San Diego, CA, USA) was used for all analyses and graphical representation.

## 3. Results

### 3.1. Expression of HSPA1A and HSPA8 Genes and Their Relationship with Apoptotic Gene Expression in Bovine Embryos In Vivo and In Vitro

**Figure 1:**
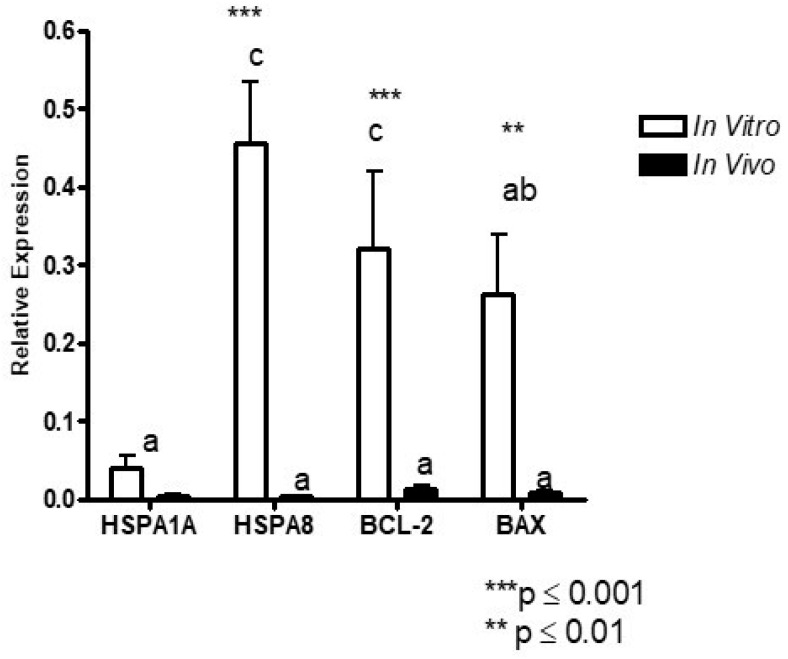
Relative expression of *HSPA1A*, *HSPA8*, *BCL-2*, and *BAX* genes in bovine embryos produced *in vitro* and *in vivo*. Gene expression levels were determined by RT-qPCR and normalized against the selected housekeeping gene using the 2^−ΔΔCt^ method. For gene expression analysis, embryos were pooled (three embryos per replicate) prior to RNA extraction. Data are presented as mean ± SEM from six independent biological replicates (*n* = 6). White bars represent *in vitro* embryos and black bars represent *in vivo* embryos. Asterisks indicate statistically significant differences between *in vitro* and *in vivo* groups (** *p* ≤ 0.01; *** *p* ≤ 0.001). Different lowercase letters above bars indicate significant differences among genes within each experimental condition (*p* < 0.05).

The basal expression levels of three of the four genes (*HSPA8, BCL-2,* and *BAX*) examined were significantly higher *in vitro* embryos compared with that of * in vivo* embryos. (** *p* ≤ 0.001/** *p* ≤ 0.01) (Figure 1). *HSPA1A* did not show a significant difference in expression between *in vitro* and *in vivo* embryos.

**Figure 2:**
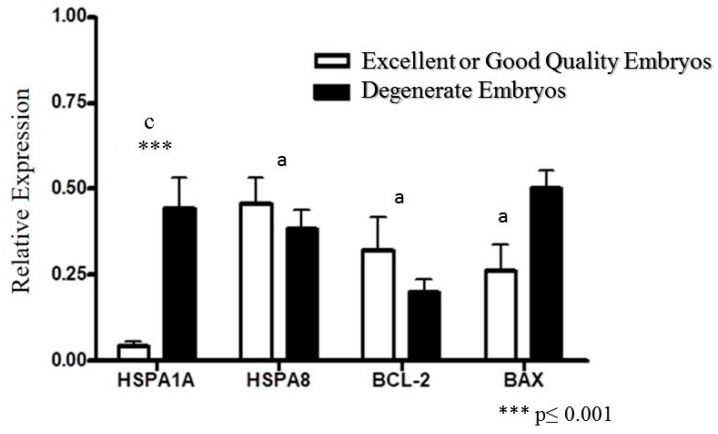
Relative expression of *HSPA1A*, *BCL-2*, and *BAX* genes according to embryo morphological quality. Gene expression was quantified by RT-qPCR and normalized to the reference gene using the 2^−ΔΔCt^ method. Embryos were classified as transferable (excellent/good quality) or degenerate based on morphological criteria. For gene expression analysis, embryos were pooled (three embryos per replicate) prior to RNA extraction. Data are presented as mean ± SEM from six independent biological replicates (*n* = 6). Asterisks indicate significant differences between quality groups (*** *p* ≤ 0.001). Different lowercase letters denote significant differences among genes within each quality category (*p* < 0.05).

The expression of the HSPA1A gene was significantly different (** *p* ≤ 0.001) between degenerate quality embryos compared with excellent or good quality embryos (transferable), and expression of the anti-apoptotic *BCL-2* gene was higher in transferable embryos compared with degenerate quality embryos. In contrast, the expression of the pro-apoptotic *BAX* gene was higher in degenerate quality embryos compared with the treated group (Figure 2).

The relative gene expression differences in the embryos supplemented with 500 ng/mL of HSC70 protein compared with the control group were evaluated. There was a six-fold up-regulation of *HSPA1A* mRNA levels, three-fold up-regulation of *HSPA8* mRNA levels, an almost three-fold up-regulation of *BCL-2* mRNA levels, and a four-fold up-regulation of *BAX* mRNA levels. Following supplementation with 1000 ng/mL, a four-fold up-regulation of *HSPA1A* mRNA levels, eight-fold up-regulation of *HSPA8* mRNA levels, an almost two-fold up-regulation of *BCL-2* mRNA levels, and an almost five-fold up-regulation of *BAX* mRNA levels compared with the control group.

The results also indicated that there was higher expression of the *HSPA1A* gene, which encodes the stress-inducible protein, HSP70, in embryos supplemented with 500 and 1000 ng/mL of HSC70 (** *p* ≤ 0.01). Supplementation with high doses (1000 ng/mL HSC70) resulted in a marked difference in *HSPA8* expression compared with the other treatments (control and 500 ng/mL; *** *p* ≤ 0.01).

**Figure 3:**
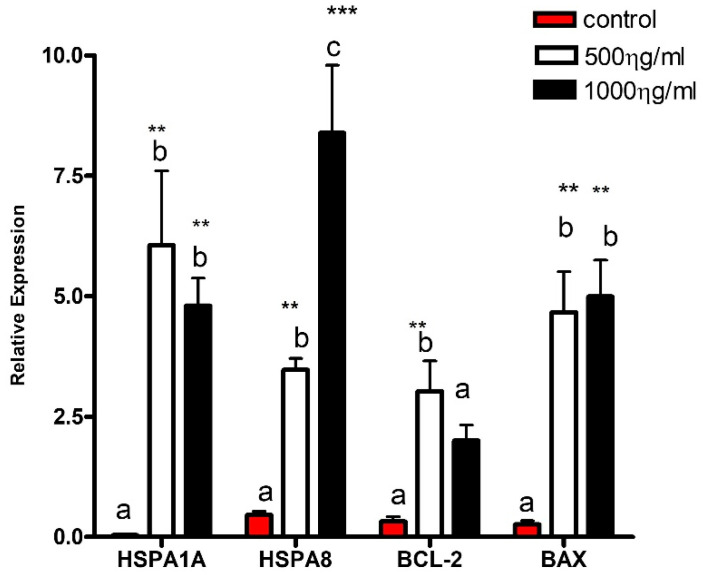
Effect of HSC70 supplementation on relative expression of *HSPA1A*, *HSPA8*, *BCL-2*, and *BAX* genes in bovine embryos produced *in vitro*. Embryos were cultured in the presence of 0 (control), 500, or 1000 ng/mL recombinant HSC70. Gene expression was determined by RT-qPCR and normalized to the reference gene using the 2−ΔΔCt method. For gene expression analysis, embryos were pooled (three embryos per replicate) prior to RNA extraction. Data are presented as mean ± SEM from six independent biological replicates (*n* = 6). Different lowercase letters above bars indicate statistically significant differences among treatment groups (*p* < 0.05). Asterisks indicate significant differences relative to the control group (** *p* ≤ 0.01; *** *p* ≤ 0.001).

The results for the apoptosis genes indicated that the anti-apoptotic *BCL-2* gene exhibited significantly higher expression at low supplementation doses (500 ng/mL, *p* ≤ 0.01); whereas the pro-apoptotic *BAX* gene showed higher expression in both treatment groups compared with the control group. (* *p* ≤ 0.01) (Figure 3).

### 3.2. Embryo Development and Morphological Assessment

Embryonic developmental parameters were evaluated to determine whether HSC70 supplementation influenced developmental competence. As shown in Table 2, 500 and 1000 ng/mL treatments significantly increased cleavage rates, the proportion of embryos reaching the ≥6-cell stage, and blastocyst formation on Days 6 and 7 compared to the control group (*p* < 0.05). Furthermore, embryos derived from the 500 ng/mL group exhibited a significantly greater total cell number at Day 7, suggesting improved morphological quality under the conditions evaluated.

Consistent with these findings, embryos supplemented with 500 ng/mL HSC70 exhibited a higher proportion of transferable-quality embryos (excellent or good), representing 57% of the total embryos obtained (18% morulae, 41% blastocysts, and 41% expanded blastocysts). In comparison, 40% of embryos in the control group were classified as regular quality (11% morulae, 28% blastocysts, 44% expanded blastocysts, and 17% hatched blastocysts). In contrast, supplementation with 1000 ng/mL resulted in a lower proportion of high-quality embryos (33%), with a higher proportion categorized as degenerate.

## 4. Discussion

HSPs are molecular chaperones that protect cells from physical or chemical damage [19,20]. They promote cell survival by regulating homeostasis [30]. In this study, we investigated the effects of supplementation with HSC70 protein on gene expression patterns and morphological parameters in bovine embryo cultures. HSPs are among the first proteins produced during embryonic development and have essential cellular functions. Although they have been extensively studied in humans and mice, information regarding the role of HSP-encoding genes in bovine embryos remains limited [31].

Identification of HSP genes that specifically contribute to embryo survival may provide an opportunity to enhance embryo protection under *in vitro* conditions and potentially improve gestation rates in cattle [32]. Embryos produced *in vitro* exhibit altered gene expression patterns reflecting stress responses to suboptimal culture environments. Oxidative stress during embryogenesis has been associated with modulation of apoptosis-related genes such as *BAX* and other stress-responsive markers [1,33,34]. Importantly, apoptosis during bovine preimplantation development is developmentally regulated. Early-stage embryos (e.g., two-cell stage) exhibit a physiological block to apoptosis, whereas activation of apoptotic pathways increases at later stages [35,36]. Moreover, expression of pro-apoptotic genes such as *BAX* and caspase-related markers has been correlated with reduced morphological quality in bovine embryos [37], supporting the use of apoptosis-related transcripts as molecular biomarkers of developmental competence.

Increased intracellular calcium has also been implicated as a mediator of apoptosis in bovine oocytes and early embryos [38], highlighting the complexity of intracellular signaling pathways regulating embryonic survival. Collectively, these studies indicate that apoptosis in preimplantation embryos is tightly regulated and context-dependent, and excessive activation under *in vitro* conditions may compromise developmental competence.

The results of this study with respect to differential expressions of HSPA8 (which encodes heat shock cognate protein 70), *BCL-2*, and *BAX* genes between embryos obtained in vivo and those produced in vitro, are consistent with the data published by Wrenzycki et al. [39]. They hypothesized that embryos produced in vitro are subject to greater stress conditions during production, such as oxygen tension and the composition of the medium used during culture. Generally, the increase in oxygen tension during in vitro embryo production increases the generation of ROS, which causes DNA damage, lipoperoxidation, and oxidative modification of proteins that increase susceptibility to proteolysis [4,16]. Therefore, antioxidants are used in oocyte maturation media and in vitro culture to improve the development of pre-implanted blastocysts [13,15,16,40,41].

Recent studies have further demonstrated that modulation of HSP70 family members can improve embryonic performance under stress conditions. For example, HSP70 supplementation improved embryo yield and quality in heat-stressed bovine oocytes [42], and HSC70 has been shown to modulate embryonic responses to thermal stress in bovine culture systems [43]. Similarly, gene expression studies in bovine embryos exposed to heat stress confirm that stress-responsive and apoptosis-related pathways are dynamically regulated depending on environmental conditions [44]. These findings support the relevance of stress-response modulation as a target for improving in vitro embryo development.

Supplementation of culture media with heat shock proteins has been shown to reduce protein aggregation and apoptosis in other cellular systems. For example, Novoselova et al. [45] demonstrated that purified HSP70 and HSC70 reduced apoptosis in cells expressing the Huntington’s disease gene by preventing aggregation of insoluble proteins. Although derived from a different biological context, these findings support the cytoprotective role of HSP70 family members.

Although several mechanistic insights into HSP70 family function derive from murine models, similar regulatory patterns have been reported in other ruminant species. In ovine and buffalo embryos, modulation of oxidative stress and apoptosis-related pathways has been associated with improved developmental competence under in vitro culture conditions [3,40]. These findings support the concept that stress-response modulation through redox balance and apoptosis regulation is conserved among ruminants.

In the present study, supplementation of embryo culture media with recombinant HSC70 was associated with gene expression patterns consistent with improved morphological embryo quality at 500 ng/mL. The elevated basal expression of *HSPA1A* and *HSPA8* observed in *in vitro* embryos likely reflects a compensatory response to culture-associated stress [46,47]. In contrast, the upregulation detected following HSC70 supplementation may represent chaperone-mediated regulatory signaling rather than stress-induced damage, since members of the HSP70/HSC70 family actively participate in apoptosis regulation and proteostasis [48,49].

These findings suggest a dose-dependent modulation of apoptosis-related gene expression rather than a purely pro- or anti-apoptotic effect. Consistent with this interpretation, members of the HSP70 family exert cytoprotective functions under stress conditions by regulating key steps in apoptotic signaling pathways [21]. HSP70 possesses well-documented anti-apoptotic properties [50,51], which align with the nearly three-fold upregulation of *BCL-2* mRNA observed in embryos supplemented with 500 ng/mL. Notably, this group also displayed improved developmental and morphological characteristics, potentially reflecting the regulatory influence of HSP70 on apoptosis-related pathways.

While HSC70 exerts well-documented anti-apoptotic effects through inhibition of *BAX* translocation to mitochondria, prevention of cytochrome c release, interference with apoptosome formation, and modulation of caspase activation [50,51], these functions operate within a tightly regulated proteostatic network [21,49]. Molecular chaperones such as HSC70 do not exert strictly linear dose-dependent effects; rather, their activity is finely balanced to maintain cellular homeostasis. Supra-physiological concentrations may disrupt this equilibrium and activate compensatory stress-responsive pathways, potentially altering the *BCL-2*/*BAX* balance [48,51]. Therefore, the increased expression of *BAX* and reduced blastocyst formation observed at 1000 ng/mL likely reflect a concentration-dependent modulation of apoptosis-related signaling rather than a simple reversal of anti-apoptotic activity or direct cytotoxicity. Given that apoptosis was assessed at the transcriptional level without functional validation, this interpretation should be considered mechanistic and associative.

Mechanistically, HSP70 interacts with both intrinsic and extrinsic apoptotic pathways at multiple levels, including inhibition of *BAX* translocation to mitochondria, prevention of cytochrome c release, interference with apoptosome formation, and suppression of caspase activation. Additionally, HSP70 modulates signaling cascades such as c-Jun N-terminal kinase (JNK), nuclear factor kappa B (NF-κB), and Akt, further contributing to its role in apoptosis regulation.

These results are consistent with the findings of Arya et al. [51], in which HSP70 family members were shown to exert context-dependent pro- or anti-apoptotic effects. In the present study, we found that supplementation with high doses (1000 ng/mL) of HSC70 resulted in a lower number of blastocysts, which was associated with increased expression of the pro-apoptotic *BAX* gene. The reduction in developmental competence observed at the highest HSC70 concentration likely reflects a concentration-associated regulatory threshold rather than direct cytotoxicity. Molecular chaperones exert tightly regulated effects on cellular proteostasis, and supra-physiological levels may disrupt homeostatic balance or activate compensatory stress signaling pathways. Members of the HSP70/HSC70 family have been shown to exert context-dependent and concentration-sensitive effects on apoptotic regulation and cell survival [48,49,51]. Excessive or dysregulated chaperone activity may alter the balance between pro- and anti-apoptotic signaling cascades rather than confer additional cytoprotection. No morphological evidence of acute degeneration was observed in the present study. Therefore, the decrease in blastocyst formation at 1000 ng/mL is more consistent with modulation of apoptosis-related signaling than with overt toxic damage.

Given that apoptosis was assessed at the transcriptional level without functional validation, this interpretation should be considered mechanistic and associated. Although the present findings support an association between HSC70 supplementation and developmental, morphological, and apoptosis-related transcriptional changes in bovine embryos produced *in vitro*, they should be interpreted with appropriate caution. Apoptosis was evaluated indirectly through gene expression analysis and morphological assessment, without functional validation at the protein or cellular level; therefore, the observed changes should be understood as molecular associations rather than direct confirmation of altered apoptotic activity. Accordingly, the apoptosis-related transcriptional profile identified in this study represents a molecular phenotype associated with HSC70 supplementation that still requires functional validation at the blastocyst level. In addition, embryos were pooled for RNA extraction to ensure sufficient RNA yield for reliable transcriptional analysis, which limited the assessment of individual biological variability. Moreover, only two HSC70 concentrations were evaluated, as these were selected on the basis of preliminary dose-screening experiments to explore biologically relevant regulatory thresholds under *in vitro* conditions rather than to define a complete dose–response curve. Since post-transfer developmental outcomes, such as implantation or pregnancy rates, were not assessed, the present results should be considered within the scope of a controlled mechanistic in vitro study. Further studies incorporating functional apoptosis assays, protein-level validation, expanded dose–response analyses, and in vivo embryo transfer outcomes will be necessary to confirm the biological and reproductive significance of HSC70 supplementation.

## 5. Conclusions

Within the limitations of this study, supplementation of bovine embryo culture media with 500 ng/mL recombinant HSC70 was associated with transcriptional modulation of apoptosis-related genes and with improved morphological embryo quality under *in vitro* conditions. In contrast, higher supplementation levels were associated with gene expression patterns indicative of enhanced stress-related signaling and reduced developmental outcomes.

The findings suggest that HSC70 exerts concentration-associated regulatory effects on developmental and apoptosis-related parameters during early bovine embryogenesis, rather than a strict dose-dependent effect on embryo viability; however, given that apoptosis was assessed at the transcriptional level without functional validation, the observed effects should be interpreted as molecular associations rather than direct confirmation of altered apoptotic activity. Further studies incorporating functional apoptosis assays, protein-level validation, and post-transfer developmental assessment are required to confirm the biological and reproductive implications of HSC70 supplementation in bovine embryo production systems.

## Figures and Tables

**Table 1:** Primer and probe sequences and amplicon sizes for target and reference genes used in this study.

Gene	Accession Number	Primer and Probe Sequences	Size (bp)
***HSPA1A***	NM_174550.1	F:CACCATTGAGGAGGTGGATTAG R:TAGCTGATGGCTGATGAAAGG P:FAM/ATGGAGACT/Zen/GTTGGGATCCAAGGC	128
***HSPA8***	NM-174345.4	F:CCAGGTTGCTGACTCTTTCA R:GGAAGACACCCACACAAGAATA P:FAM/TGCAGTTGG/Zen/CATTGATCTTGGCAC	96
***Invitro BAX***	NM_173894.1	F:CGAGTTGATCAGGACCATCAT R:ATGTGGGTGTCCCAAAGTAG P:HEX/TCGAAGGAA/Zen/GTCCAATGTCCAGCC	579
***BCL-2***	NM_001166486.1	F:GATTTCTCCTGGCTGTCTCTG R:GCCTGTGGGCTTCACTTAT P:FAM/TTGCATCAC/Zen/CCTGGGTGCCTAT	792
***GAPDH***	NM_001034034.2	F:TGAGATCAAGAAGGTGGTGAAG R:GCATCGAAGGTAGAAGAGTGAG P:FAM/CCAGGTTGT/Zen/CTCCTGCGACTTCAA	82

**Table 2:** Effect of HSC70 supplementation on developmental competence of *in vitro*-produced bovine embryos.

Treatment (ng/mL)	n (Embryos)	≥6-Cell Embryos (%)	Cleavage Rate (%)	Blastocysts Day 6 (%)	Blastocysts Day 7 (%)	Total Cell Number (Day 7)
0 (Control)	384	70.27 ± 3.9 ᵃ	51.03 ± 7.7 ᵃ	10.81 ± 1.5 ᵃ	15.98 ± 5.0 ᵃ	132.81 ± 17.8 ᵃ
500	387	80.64 ± 4.6 ᵇ	65.81 ± 5.0 ᵇ	17.48 ± 3.5 ᵇ	24.92 ± 1.9 ᵇ	127.08 ± 18.4 ᵃ
1000	374	82.72 ± 3.2 ᵇ	66.23 ± 4.6 ᵇ	19.34 ± 1.9 ᵇ	28.25 ± 4.9 ᵇ	132.81 ± 17.8 ᵃ

Notes: Values are expressed as mean ± SEM from six independent replicates (n = 6). Different superscript letters within the same column indicate statistically significant differences (*p* < 0.05) as determined by one-way ANOVA followed by Tukey’s multiple comparison test.

## Data Availability

The data presented in this study are available on request from the corresponding author due to institutional restrictions.
